# Retinal Degeneration After First-Ever Optic Neuritis Helps Differentiate Multiple Sclerosis and Neuromyelitis Optica Spectrum Disorder

**DOI:** 10.3389/fneur.2019.01076

**Published:** 2019-10-10

**Authors:** Nam-Hee Kim, Ho Jin Kim, Cheol-Yong Park, Kyoung Sook Jeong

**Affiliations:** ^1^Department of Neurology, Dongguk University Ilsan Hospital and Dongguk University-Seoul, Graduate School of Medicine, Goyang, South Korea; ^2^Department of Neurology, National Cancer Center, Goyang, South Korea; ^3^Department of Ophthalmology, Dongguk University Ilsan Hospital and Dongguk University-Seoul, Graduate School of Medicine, Goyang, South Korea; ^4^Department of Occupational and Environmental Medicine, Hallym University Sacred Heart Hospital, Anyang, South Korea

**Keywords:** optical coherence tomography, neuromyelitis optica spectrum disorder, multiple sclerosis, optic neuritis, retinal degeneration

## Abstract

**Objective:** Differentiation between neuromyelitis optica spectrum disorder (NMOSD) and multiple sclerosis (MS) in the early phase is challenging but crucial for treatment and prognosis.

**Methods:** We performed a prospective cross-sectional study to discriminate NMOSD from MS by evaluating retinal degeneration in optical coherence tomography (OCT) after a first-ever optic neuritis (ON) episode. Seventy-three NMOSD patients and 38 MS patients with ON at least 3 months prior were assessed by OCT, best-corrected visual acuity (VA), and 2.5% low-contrast VA. Multivariate linear regression models were used for comparisons. Receiver operating characteristic curves and Youden index were used for determining the discriminative value of retinal nerve fiber layer thickness (RNFL) and VA in distinguishing NMOSD from MS.

**Results:** Among eyes with retinal degeneration after a first-ever ON episode (*n* = 93), NMOSD eyes (*n* = 60) presented thinner RNFL (*p* < 0.001) and worsened VA (*p* < 0.001) relative to MS eyes (*n* = 33). Furthermore, a RNFL thinner than 78.9 μm had a specificity of 93.9% for NMOSD; combined with a VA of <0.4 decimal, these characteristics provided 100% specificity for NMOSD.

**Conclusions:** The first-ever ON eyes showed more severe retina degeneration in patients with NMOSD than MS, which could establish a cut-off of RNFL thickness and VA to distinguish NMOSD from MS in the early phase.

## Introduction

The early and accurate distinction of neuromyelitis optica spectrum disorders (NMOSD) from multiple sclerosis (MS) is important, as the prognosis and treatments for these two diseases are very different (1, 2). Although testing for the aquaporin-4 antibody has facilitated this differentiation, an accurate diagnosis remains challenging, particularly among those with seronegative NMOSD (2).

MS and NMOSD have often been reported to occur with optic neuritis (ON) (2). Axonal injury occurs to a greater extent in NMOSD, whereas the demyelination associated with MS occurs with some preservation of axons (1). Therefore, the visual prognosis is worse in those with NMOSD (1, 3).

Optical coherence tomography (OCT) has been used to measure the thickness of retinal nerve fiber layer (RNFL) and the macular volume (MV) in patients with MS and NMOSD (2, 4). Over the years, several studies have suggested that by using OCT, RNFL, and MV can be used to detect axonal loss in MS and monitor treatment efficacy (2, 5, 6). The RNFL consists of retinal ganglion cell axons that coalesce to form the optic nerve (7). MV is determined by the number of retinal ganglion cell bodies, photoreceptors, and other cell types (8). A significant decrease in RNFL thickness and MV has been known to occur in the eyes of patients with MS, with and without a history of ON (2). Moreover, these abnormalities were found to correlate with brain atrophy in MS (9).

The literature has shown a significant reduction in RNFL thickness after ON among patients with NMOSD, much more than among those with MS (2, 7). Thus, RNFL thickness has been suggested to be useful in discriminating between NMOSD and MS (7, 10). However, in patients with a history of multiple ON episodes, the discrimination using RNFL thickness may not be easy. Cumulative damage from repeated attacks on the optic nerve may cause an extreme reduction in RNFL thickness even among patients with MS, to as severe a degree as in NMOSD (7, 11, 12). Despite the critical problem of discriminating between these two conditions using OCT, previous studies have focused solely on the differences after considering a history of ON regardless of the number of episodes. Thus, assessing eyes with a single ON episode can demonstrate disease-specific pathology in the optic nerve, averting changes obscured by multiple episodes of ON.

Therefore, by studying eyes with the first-ever ON episode, we sought to determine whether RNFL thickness could be used as a diagnostic tool to differentiate between NMOSD and MS. If possible, we also hoped to establish a cut-off value of RNFL thickness for distinguishing between NMOSD and MS in the early stage of disease.

## Materials and Methods

### Study Population and Clinical Data

This observational and cross-sectional study was performed according to the tenets of the Declaration of Helsinki, and was approved by the institutional review board of Dongguk University Ilsan Hospital. All subjects provided informed written consent. We recruited patients with NMOSD who were seropositive for the aquaporin-4 antibody defined by the revised 2006 diagnostic criteria of Wingerchuk (13) and recruited patients with MS who met the 2010 McDonald criteria (14). Subjects with diabetes, a history of ocular injury, glaucoma, or other ophthalmologic disorders were excluded. In addition, patients within 3 months of acute ON were excluded to minimize the effect of ON-related edema on OCT measurements (15).

### Optical Coherence Tomography

Retinal imaging was performed using a time-domain OCT (Stratus OCT-3) with OCT 4.0 software (Carl Zeiss Meditec Inc., Dublin, CA). RNFL thickness was measured by the “Fast RNFL Thickness” protocol and the MV was obtained using the “Fast Macular Thickness” scan. Scans with signal strength <7 or with artifact were excluded from the analysis. For eyes with extremely poor visual function (unable to fixate), OCT scans were acquired with external fixation of the fellow eye. RNFL thickness for the temporal, superior, nasal, and inferior quadrants and the overall mean of these quadrants as well as the total MV were obtained by OCT as we previously described (16). The normal range of mean RNFL is 82–110 μm.

### Visual Function

Best-corrected visual acuity (BCVA) with 100% contrast (high contrast visual acuity, HCVA) was measured using the standard Snellen chart. Sloan letter charts of 2.5% contrast (Precision Vision, La Salle, IL) were used for low-contrast visual acuity (LCVA). Visual acuity (VA) was expressed using a decimal scale but was transformed to the logarithm of the minimum angle of resolution (logMAR) for statistical analyses.

### Statistical Analysis

Comparisons between groups were performed using the Student *t*-test or Mann–Whitney test considering normality and the properties of the variables. OCT measures and visual functions were compared between the groups using multivariate linear regression models adjusting for age and disease duration. Pearson's and Spearman's rank order correlations were used to assess correlations among visual functions, the expanded disability status scale (EDSS), and OCT measurements. Receiver operating characteristic (ROC) curves were used to determine the discriminative value of OCT and VA in eyes with a first-ever ON episode to distinguish between NMOSD and MS. The cut-off values were calculated using the Youden index as the points with the best sensitivity-specificity balance (17). Statistical significance was defined as *p* < 0.05. Statistical analysis was performed using SPSS version 20 (IBM, NY, USA).

## Results

Seventy-three patients with seropositive NMOSD and 38 with relapsing remitting MS were analyzed. Subjects with NMOSD were older and had a higher ratio of female participants, higher EDSS score, longer disease duration, and higher number of ON episode than MS patients. Patients' demographic and clinical characteristics are shown in Table 1.

**Table 1:** Demographic characteristics of patients with neuromyelitis optica spectrum disorders or multiple sclerosis.

	**Neuromyelitis optica spectrum disorder**	**Multiple sclerosis**	***p*-value**
Number of subjects	73	38	
Mean age, years (SD)	39.4 (12.0)	35.2 (10.0)	0.026
Gender, F:M	63:10	24:14	<0.001
Median EDSS (ranges)	3.5 (1.0–9.0)	1.5 (0–7.5)	<0.001
Mean disease duration, years (SD)	6.5 (4.5)	4.5 (3.8)	<0.001
Number of optic nerves affected by ON (%)	101 (69)	38 (50)	0.003
Median episodes of ON (ranges)	1.0 (0–12)	0 (0–11)	0.003
NMO-IgG positive (%)	100	0	<0.001

*MS, multiple sclerosis; NMO, neuromyelitis optica spectrum disorder; ON, optic neuritis; EDSS, Expanded Disability Status Scale*.

Eyes were categorized into six groups: MS without ON episodes (*N* = 38), MS with a single ON episode (*N* = 33), MS with multiple ON episodes (*N* = 5), NMOSD without ON episodes (*N* = 45), NMOSD with a single ON episode (*N* = 60), and NMOSD with multiple ON episodes (*N* = 41) (Table 2). In the multiple ON episode group, the number of ON episode, age, or disease duration did not differ between patients with NMOSD or MS. Among those with a single ON episode, there was no difference in age or disease duration between patients with MS and NMOSD.

**Table 2:** Retinal nerve fiber thicknesses and visual functions for multiple sclerosis and neuromyelitis optica spectrum disorder.

	**Neuromyelitis optica spectrum disorder**			**Multiple sclerosis**			***p*-value**
	**Unaffected**	**Affected**		**Unaffected**	**Affected**		
		**Single ON**	**Multiple ON**		**Single ON**	**Multiple ON**	**Single ON**
	**(*N* = 45)**	**(*N* = 60)**	**(*N* = 41)**	**(*N* = 38)**	**(*N* = 33)**	**(*N* = 5)**	**NMOSD vs. MS**
**RNFL thickness**, **μm, mean (SD)**							
Average	105.5 (11.9)	76.0 (24.8)	49.9 (16.4)	102.7 (11.9)	95.2 (14.0)	77.4 (11.4)	<0.001
Temporal	79.0 (15.7)	57.0 (21.8)	40.6 (14.2)	73.9 (17.3)	71.4 (24.8)	44.2 (12.2)	0.008
Inferior	137.9 (19.3)	98.9 (36.3)	58.4 (27.1)	133.3 (16.3)	123.2 (20.8)	99.4 (13.2)	<0.001
Nasal	78.6 (16.3)	56.3 (18.1)	43.7 (11.2)	78.6 (14.6)	68.8 (12.4)	65.2 (8.9)	<0.001
Superior	126.7 (18.6)	91.9 (34.9)	57.0 (21.9)	124.5 (17.4)	117.2 (18.8)	100.4 (20.0)	<0.001
**Macular volume, mm**^**3**^**, mean (SD)**							
	6.77 (0.43)	6.07 (0.77)	5.50 (0.43)	6.75 (0.36)	6.58 (0.37)	6.27 (0.41)	<0.001
**Visual acuity, LogMAR, median (mean)**							
100% contrast	0.10 (0.26)	0.75 (0.93)	1.60 (1.63)	0.20 (0.35)	0.10 (0.33)	1.30 (1.10)	<0.001
2.5% contrast	0.60 (0.81)	1.80 (1.24)	1.80 (1.74)	0.50 (0.60)	0.80 (1.02)	0.80 (1.00)	0.118

*MS, multiple sclerosis; NMOSD, neuromyelitis optica spectrum disorder; ON, optic neuritis; EDSS, Expanded Disability Status Scale; RNFLT, retinal nerve fiber layer thickness; p-values after adjusting age and disease duration*.

Comparisons of OCT and visual function measures between eyes with MS and NMOSD according to the number of ON episodes

**Figure 1:**
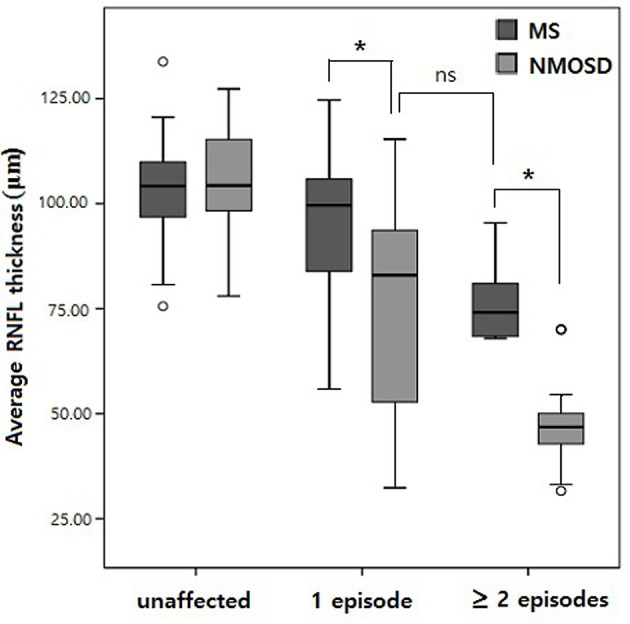
Box plot comparing retinal nerve fiber layer (RNFL) thickness by the number of optic neuritis episodes. *p < 0.01; ns, not significant.

RNFLs in affected eyes were thinner in all four quadrants in NMOSD eyes relative to MS eyes (*p* < 0.001 for each, Table 2), whereas RNFLs in unaffected eyes did not differ between patients with NMOSD and MS, except for a thinner temporal quadrant in MS eyes (*p* = 0.009). When comparing RNFL thickness among eyes with a single ON episode, the RNFLs on average as well as all quadrants were thinner in NMOSD eyes relative to MS eyes (*p* < 0.001 for each; Table 2, Figure 1). Among eyes from patients with multiple ON episodes, RNFLs on average as well as all quadrants except the temporal quadrant were thinner in NMOSD eyes than in MS eyes (*p* < 0.001 for nasal, superior, and inferior quadrants; *p* = 0.595 for the temporal quadrant; see Table 2). In addition, the thickness of RNFLs in NMOSD eyes with a single ON episode did not differ from those of MS eyes with multiple ON episodes, suggesting the importance of controlling for the number of ON episodes when comparing eyes of these different diseases (Table 2).

The total MV in affected eyes of NMOSD was reduced compared to those with MS (*p* < 0.001; Table 2), whereas there was no difference in MV between unaffected eyes of NMOSD and those with MS. In each group categorized with the number of ON episodes, the MV was significantly reduced among eyes with NMOSD than in those with MS (*p* < 0.001 for groups with a single ON episode, *p* = 0.001 for groups with multiple ON episodes, Table 2). However, there was no difference in the MV between NMOSD eyes with a single ON episode and MS eyes with multiple episodes of ON.

HCVA and LCVA were worse in the affected eyes of NMOSD compared to those of MS (*p* < 0.001), whereas those were not different between unaffected eyes of NMOSD and unaffected eyes of MS (Table 2). When comparing the eyes with a single ON episode, HCVA was significantly worse in NMOSD relative to MS (*p* < 0.001). LCVA did not differ between NMOSD and MS. HCVA and LCVA in NMOSD eyes with a single ON episode did not differ from MS eyes with multiple episodes of ON.

Among patients with a history of a single unilateral ON, the difference in RNFL thickness between both eyes was significantly larger among patients with NMOSD (*N* = 10, 24.1 ± 18.8 μm) relative to patients with MS (*N* = 8, 10.3 ± 4.1 μm) (*p* = 0.004).

Discrimination between eyes with MS and NMOSD after a first-ever ON.

After a first episode of ON, RNFL thickness, MV, and HCVA in NMOSD were significantly worse compared to those of MS (*p* < 0.001; Table 2).

In ROC curve analyses, the average RNFL thickness “cut-off” value was 78.9 μm with 93.9% specificity and 45.0% sensitivity for discrimination of NMOSD from MS. And HCVA “cut-off” value was 0.40 in LogMAR (0.40 in decimal) with 72.7% specificity and 65.0% sensitivity for discrimination of NMOSD from MS. Furthermore, the cut-off value of RNFL thickness <78.9 μm with HCVA <0.40 decimal showed 100% specificity for NMOSD (Table 3).

**Table 3:** Receiver operating characteristic curve analysis of the retinal nerve fiber layer thickness, the macular volume, and visual acuity in those with a first-ever ON episode.

	**AUC**	***p*-value**	**Cut-off value**	**Specificity for NMOSD**	**Sensitivity for NMOSD**	**J-index**
**RNFL thickness**, **μm**						
Average	0.704	0.001	78.9	0.94	0.45	0.39
Temporal	0.673	0.006	50.0	0.85	0.45	0.30
Inferior	0.699	0.002	123.5	0.58	0.77	0.34
Nasal	0.717	0.001	58.5	0.81	0.53	0.35
Superior	0.734	<0.001	101.0	0.88	0.53	0.41
MV, mm^2^	0.722	<0.001	6.3	0.85	0.45	0.37
**VA, decimal**						
100% contrast	0.704	0.001	0.4	0.73	0.65	0.38

*RNFL, retinal nerve fiber layer; MV, macular volume; VA, visual acuity; LogMAR, Logarithm of the minimum angle of resolution; NMOSD, neuromyelitis optica spectrum disorder; AUC, area under the Receiver operating characteristics curve; J-index, Youden index*.

RNFL thickness in a first-ever ON episode correlated with disability and disease duration.

When evaluating all 93 eyes with a first-ever ON episode, RNFL thickness demonstrated strong negative correlations with HCVA, 2.5% LCVA, EDSS, and disease duration (Table 4). Among the MS eyes with a first-ever ON episode (*N* = 33), RNFL thickness was significantly correlated with 2.5% LCVA, EDSS, and disease duration (Table 4). In the analysis of the NMOSD eyes with a first-ever ON episode (*N* = 60), RNFL thickness was significantly correlated with HCVA, 2.5% LCVA, EDSS, and disease duration (Table 4).

**Table 4:** Correlations among the RNFL thickness, HCVA, LCVA, EDSS score, and disease duration.

	**HCVA**	**LCVA**	**EDSS**	**Disease duration**
**All first-ever ON eyes (*****N*** **= 93)**				
RNFLT	*r* = −0.643 (*p* < 0.001)	ρ = −0.471 (*p* < 0.001)	*r* = −0.426 (*p* < 0.001)	*r* = −0.324 (*p* = 0.002)
**First-ever ON eyes of MS (*****N*** **= 33)**				
RNFLT	*r* = −0.034 (*p* = 0.852)	ρ = −0.374 (*p* = 0.035)	*r* = −0.376 (*p* = 0.031)	*r* = −0.374 (*p* = 0.032)
**First-ever ON eyes of NMOSD (*****N*** **= 60)**				
RNFLT	*r* = −0.691 (*p* < 0.001)	ρ = −0.523 (*p* < 0.001)	*r* = −0.385 (*p* = 0.002)	*r* = −0.325 (*p* = 0.011)

*r, Pearson's r; ρ, Spearman's ρ; RNFLT, retinal nerve fiber layer thickness; HCVA, high contrast visual acuity; LCVA, low contrast visual acuity; EDSS, Extended Disability Status Score; ON, optic neuritis; p-values are given brackets*.

## Discussion

Multiple episodes of ON in the advanced disease state can result in cumulative and extensive axonal damage as well as visual disturbance (18, 19) and may thus attenuate differences between NMOSD and MS. Our study confirmed that the NMOSD eyes with a single ON attack showed the thinning of the RNFL and visual disturbances similar to that of MS eyes with multiple ON attacks, supporting the idea that cumulative damage of repeat attacks can mask disease-specific effects on optic nerve. Previous studies using OCT have compared affected or unaffected eyes for both diseases without considering the number of ON episodes (2, 7, 20). Thus, a study examining eyes with a first-ever ON episode may be more useful in understanding disease-specific retinal pathology.

We found that after a single episode of ON, thinning of the peripapillary RNFL was significantly greater in the eyes of NMOSD compared with those of MS and was paralleled by poorer HCVA. In a systemic review and meta-analysis, the inter-eye RNFL difference was 30.98 μm in NMOSD and 9.87 μm in MS between eyes with or without ON, in which ON included both single and multiple episodes (21). In our study, 29.5 and 7.5 μm in RNFL thickness were decreased after single ON attack in NMOSD and MS, respectively. These differences are consistent with findings reported by previous seminal studies using OCT (2, 7, 10, 20, 22).

Furthermore, we could calculate cut-off values for discriminating NMOSD from MS among those with a first-ever ON episode. We chose cut-off values for RNFL thickness and HCVA excluding MV, because MV is strongly associated with RNFL which consists one of the layers in the macula. Accordingly, we extracted the average cut-off value of 78.9 μm for RNFL thickness and 0.4 decimal for HCVA. Using the average RNFL thickness <78.9 μm, the specificity was 93.9% for discriminating NMOSD from MS. RNFL thickness of <78.9 μm with HCVA of <0.4 decimal showed 100% specificity for NMOSD. These results support the theory that RNFL thickness and HCVA could be used as tools for differential diagnosis of MS and NMOSD. In a study by Peng et al. they suggested cut-off value of inferior nasal RNFL thickness in NMO-ON as ≤46.5 μm for discrimination with healthy control and the specificity was 57.5% (23). Our study suggests the cut-off value to distinguish between NMOSD and MS after the first-ever ON with an average of 78.9 μm for all quadrants of RNFL thickness.

Interestingly, the association of structural retinal damage and impairment of visual function was more apparent in patients with NMOSD relative to patients with MS. In a first-ever ON episode, RNFL thickness and HCVA had a significant correlation among patients with NMOSD (*r* = −0.643, *p* < 0.001) but not among patients with MS (*r* = −0.034, *p* = 0.852). Furthermore, a significant correlation between EDSS and RNFL thickness among patients with NMOSD was confirmed in our study with the relatively large cohort, while there has previously been controversy about the association of RNFL thickness and EDSS in this patient population (2, 3, 8, 11, 24, 25).

We compared the seropositive NMOSD patients with MS patients to avoid controversy over the diagnosis of NMOSD. However, given the recently published papers, the results of this study may also be applicable to the seronegative NMOSD patients (26, 27).

In summary, this study revealed that the RNFL thickness among those with a first-ever ON episode was useful in understanding disease-specific retinal pathology. RNFL thickness and HCVA after a first-ever ON was significantly different among eyes with NMOSD than eyes with MS. We suggest that RNFL thickness with visual function among those with a first-ever ON episode will help discriminate NMOSD from MS in the early stage of diseases, by using cutoff values for the average RNFL thickness and HCVA.

## Data Availability Statement

The datasets generated for this study are available on request to the corresponding author.

## Ethics Statement

The studies involving human participants were reviewed and approved by Institutional review board of Dongguk University Ilsan Hospital. The patients/participants provided their written informed consent to participate in this study.

## Author Contributions

N-HK, HK, and C-YP contributed to the design of the work and interpreted the results. N-HK and KJ executed the analyses and wrote the draft of the paper. HK and C-YP supervise the work. KJ revised the manuscript. All authors read and approved the final manuscript.

### Conflict of Interest

The authors declare that the research was conducted in the absence of any commercial or financial relationships that could be construed as a potential conflict of interest.
